# A Rare Case of Postpartum Portal Vein Thrombosis in a Young Patient With Heterozygous Prothrombin G20210A Mutation

**DOI:** 10.7759/cureus.78544

**Published:** 2025-02-05

**Authors:** Fatima I Hsayan, Nour T Matar, Jeannette G Sarkis, Violette E Issa, Antoine Abou Rached

**Affiliations:** 1 Internal Medicine, Lebanese University Faculty of Medical Sciences, Beirut, LBN; 2 Gastroenterology, Lebanese University Faculty of Medical Sciences, Beirut, LBN

**Keywords:** epigastric pain syndrome, portal thrombosis, postpartum, postpartum fever, venous thromboembolism (vte)

## Abstract

Portal vein thrombosis (PVT) is most commonly associated with cirrhosis due to the reduced blood flow through the liver. However, its occurrence in non-cirrhotic individuals is rare and typically linked to hypercoagulable states. The postpartum period is a recognized hypercoagulable state, yet PVT in this context remains uncommon.

We report a rare case of PVT in a 25-year-old female patient diagnosed with a heterozygous prothrombin G20210A mutation, presenting six weeks after a cesarean section with recurrent epigastric pain, fever, and elevated cholestatic liver enzymes. Multifactorial etiologies, including the hypercoagulable postpartum period, prolonged bed rest, and the presence of the prothrombin G20210A mutation, contributed to the development of venous thrombosis.

This case underscores the synergistic effect of genetic predisposition and pregnancy-related changes on thrombotic risk. It also highlights the importance of maintaining a high index of suspicion for PVT in postpartum females presenting with epigastric pain and fever. Our patient was successfully managed with anticoagulation therapy, which resolved her symptoms and normalized her liver function tests. This case raises critical questions about the long-term management of thrombotic risk in similar patients.

## Introduction

Portal vein thrombosis (PVT) refers to the narrowing or complete obstruction of the portal vein, its intrahepatic branches, or the proximal splenic and mesenteric veins due to thrombus formation [1,2]. The pathogenesis of PVT is multifactorial, often associated with Virchow's triad: venous stasis, vascular damage, and hypercoagulability [3]. These factors may be inherited, such as deficiencies in protein S, protein C, or antithrombin, or acquired, including local and systemic inflammation, liver disease, pancreatitis, malignancy, and other conditions [2,3].

PVT is particularly prevalent in patients with liver disease, with a higher risk in decompensated cirrhosis compared to preserved liver function [3]. In individuals with normal liver function, PVT is less common and often linked to thrombophilic disorders [2,3]. Its overall incidence is estimated at 2-4 cases per 100,000 population, with a higher prevalence in patients with portal hypertension secondary to cirrhosis, particularly in developing countries [3].

The clinical presentation of PVT varies. Acute PVT often manifests abruptly with symptoms such as severe abdominal pain, distension, diarrhea, and fever, whereas chronic PVT presents more insidiously, with splenomegaly, ascites, or no symptoms at all [1,2]. Pregnancy and the postpartum period are well-established risk factors for thromboembolic events, with the risk of venous thromboembolism (VTE) increasing four- to fivefold compared to non-pregnant women, particularly within the first six weeks postpartum [4,5].

Here, we report the case of a young woman who developed segmental portal vein thrombosis six weeks after a cesarean section. This case highlights the interplay of genetic predisposition and postpartum factors, emphasizing the importance of tailored anticoagulation strategies.

## Case presentation

A 25-year-old woman, a non-smoker with no known allergies, presented to the emergency department with acute epigastric pain, not exacerbated by eating, with multiple episodes throughout the day, high-grade fever (40°C), and one episode of non-bloody, non-bilious vomiting. She had undergone a cesarean section six weeks prior and was not on any medications. Physical examination revealed a soft abdomen with negative Murphy's and McBurney's signs. Her vital signs were stable, except for the high-grade fever.

Laboratory findings showed leukocytosis with white blood cell (WBC) count of 16,000/mm³ (reference range: 4,000-10,000/mm³), neutrophils at 81%, elevated C-reactive protein (CRP) of 86.4 mg/dL (reference range: 0-0.7 mg/dL), and a cholestatic pattern of liver enzyme elevation including gamma-glutamyl transferase (GGT) of 335 U/L (reference range: 10-49 U/L), alkaline phosphatase (ALP) of 254 U/L (reference range: 64-306 U/L), total bilirubin of 1.6 mg/dL (reference range: 0-1.4 mg/dL), direct bilirubin of 0.5 mg/dL (reference range: 0-0.4 mg/dL), serum glutamic-oxaloacetic transaminase (SGOT) of 131 U/L (reference range: 10-37 U/L), and serum glutamic pyruvic transaminase (SGPT) of 68 U/L (reference range: 10-45 U/L). Lipase and amylase levels were normal. Abdominal ultrasound ruled out gallstones and bile duct dilation. Viral serologies (Epstein-Barr Virus (EBV), cytomegalovirus (CMV), and herpes simplex virus (HSV)) were requested but not performed as the patient left the hospital against medical advice.

**Figure 1:**
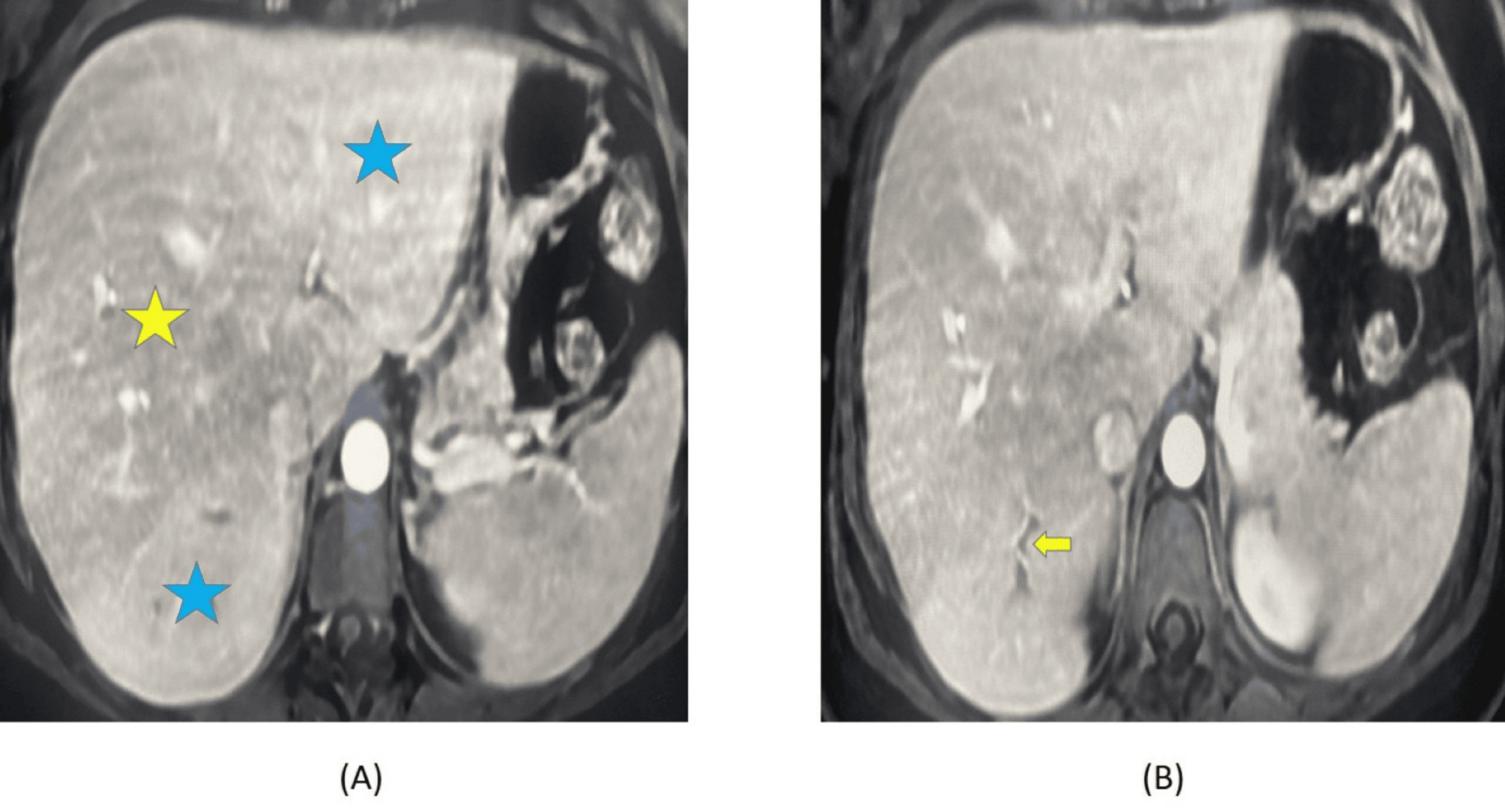
Image (A) demonstrated a perfusion defect marked by a difference in liver parenchyma. The hypointense liver parenchyma in the region of segment VII (yellow star) contrasts with the well-perfused liver parenchyma (blue stars). Image (B) showed a segmental portal vein thrombosis in segment VII (yellow arrow) that results in a perfusion disorder.

Nine days later, she presented to another facility with recurrent epigastric pain and high-grade fever(40°C). Repeated tests revealed leukocytosis with a WBC count of 14,300/mm³, elevated CRP of 17.14 mg/dL, and further increases in liver enzymes with GGT of 890 U/L, ALP of 882 U/L, SGPT of 239 U/L, and SGOT of 78 U/L. Magnetic resonance cholangiopancreatography (MRCP) was performed and revealed segmental portal vein thrombosis in segment VII and gallstones (Figure 1).

The patient was started on rivaroxaban (20 mg daily), and a hypercoagulable state workup was initiated. Results identified a heterozygous prothrombin gene mutation (G20210A) with normal protein C, protein S, and antithrombin levels. Factor V Leiden, JAK2 mutations, and antiphospholipid antibodies were absent (Table 1).

**Table 1:** Hypercoagulable workup

Hypercoagulable workup	Results	Reference values
Antithrombin activity	84%	Normal: >75%
Protein C activity	91%	Normal: >70%
Protein S activity	90%	Normal: >55%
PCR factor 5 Leiden	Absence of genetic mutation	-
PCR prothrombin gene	Presence of heterozygous mutation	-
JAK2 mutation	Absence of genetic mutation	-
Anticardiolipin IgG	2.2 AU/mL	Negative: <20 AU/mL, positive: >20 AU/mL
Anticardiolipin IgM	0.5 AU/mL	Negative: <20 AU/mL, positive: >20 AU/mL

After one month of anticoagulation with rivaroxaban, her symptoms resolved, and there has been no recurrence of epigastric pain or fever, with normalization of liver enzymes (SGPT: 28 U/L, SGOT: 29 U/L, GGT: 70 U/L, ALP: 83 U/L, total bilirubin: 0.5 mg/dL, and direct bilirubin: 0.1 mg/dL).

## Discussion

Our 25-year-old female patient presented with epigastric pain, fever, and elevated cholestatic liver enzymes with symptoms that initially suggested viral hepatitis, gallstones, or choledocholithiasis. However, imaging revealed segmental portal vein thrombosis (PVT) in the medial part of segment VII, accompanied by gallstones. Hypercoagulable state workup identified a heterozygous prothrombin gene mutation (G20210A) as a contributing factor. Following one month of anticoagulation therapy with rivaroxaban, her symptoms resolved, and her liver function tests normalized.

The postpartum period is a well-known hypercoagulable state, yet hepatic vein thrombosis, including PVT, remains rare and likely underreported. A thorough PubMed search using the terms "postpartum," "pregnancy," "hepatic thrombosis," and "hepatic vein thrombosis" yielded only three cases of peripartum PVT in the literature, to which we add our case.

Among the three cases identified, only one closely paralleled our patient's presentation. This case described a young, previously healthy woman who developed non-cirrhotic PVT after a C-section and strict preterm bed rest [6].

The other two cases involved distinct etiologies. The first described a postpartum patient with thrombotic thrombocytopenic purpura (TTP) and Budd-Chiari syndrome following platelet transfusions and plasmapheresis [7]. The second involved a previously undiagnosed pancreatic cancer patient who developed hepatic vein thrombosis and a stroke during pregnancy [8].

In our patient, multiple risk factors compounded her susceptibility to venous thromboembolism (VTE). Additionally, her recent C-section likely prolonged her recovery, necessitating extended bed rest [9]. The discovery of her heterozygous G20210A genetic mutation further heightened her risk.

Heterozygous carriers of the G20210A mutation are generally not categorized as high-risk for requiring prophylactic anticoagulation during pregnancy, unlike homozygous carriers. Studies estimate the risk in heterozygous patients to be approximately one in 400, although this risk escalates in the presence of additional factors [10,11].

Our case underscores that genetic predisposition alone should not be the sole determinant for prophylactic anticoagulation during pregnancy. This is corroborated by a meta-analysis by Ziakas et al., which examined thrombophilia and VTE in pregnancy [12].

Notably, a limitation in this case was the inability to rule out lupus anticoagulant, as the test was ordered but not performed. Additionally, the patient's decision to leave against medical advice during her initial presentation delayed the diagnosis, resulting in a recurrence of symptoms and worsening laboratory findings.

## Conclusions

This case emphasizes the importance of maintaining a high index of suspicion for PVT in postpartum women presenting with epigastric pain and fever, particularly within the first six weeks postpartum. Testing for hypercoagulable states is critical, even during the puerperium, as studies suggest a synergistic effect between pregnancy-induced changes and genetic mutations such as factor V Leiden or prothrombin G20210A mutation.
